# Persistent Neutrophilic Meningitis in an Immunocompetent Patient after Basilar Skull Fracture: Case Report

**DOI:** 10.1186/1471-2334-11-136

**Published:** 2011-05-19

**Authors:** Jaime S Green, Shira R Abeles, Daniel Z Uslan, Sanjay R Mehta

**Affiliations:** 1 Department of Medicine, Division of Infectious Diseases, University of California, San Diego, California, USA; 2 Department of Medicine, Division of Infectious Diseases, University of California, Los Angeles, California, USA

## Abstract

**Background:**

Persistent neutrophilic meningitis is an unusual form of chronic meningitis that is defined as clinical meningitis with a neutrophilic pleocytosis that persists for greater than 7 days despite empiric antimicrobial therapy. Although numerous disease processes can cause this syndrome, the majority of cases are due to opportunistic pathogens infecting immunocompromised hosts.

**Case Presentation:**

A 47 year-old female presented after basilar skull fracture with persistent neutrophilic meningitis unresponsive to empiric broad-spectrum antibiotics. After more than weeks of intensive therapy, 4 hospitalizations and 3 relapses, *Nocardia cyriacigeorgica *was identified from cerebral spinal fluid. Induction therapy was begun with Ceftriaxone and trimethoprim-sulfamethoxazole (TMP-SMX) for 6 weeks followed by therapy with TMP-SMX and doxycycline for one year. The patient made a complete recovery without sequelae.

**Conclusions:**

Due to the difficulty in obtaining a microbiologic diagnosis, appropriate treatment in cases of persistent neutrophilic meningitis is often delayed leading to morbidity, This case highlights a number of the unique features of *Nocardia *meningitis and the importance of considering *Nocardia *infection as a cause of persistent neutrophilic meningitis even in immunocompetent patients.

## Background

Persistent neutrophilic meningitis is a syndrome defined by: clinical meningitis (headache, altered sensorium, fever and/or meningismus) with neutrophilic cerebrospinal fluid (CSF) pleocytosis (differential containing >50% neutrophils), elevated protein and hypoglycorrhachia that persists for greater than 7 days despite appropriate empiric antimicrobial treatment [1]. Numerous infectious and non-infectious etiologies have been identified as causes of this syndrome including: saprophytic intracellular bacteria (eg. *Nocardia, Actinomyces *and *Brucella*), mycobacteria, fungi (eg. *Candida spp., Aspergillus spp*., zygomycetes, endemic fungi), connective tissue disease (eg. Lupus, adult Still's disease [2], central nervous system (CNS) vasculitides), chemical meningitis, intracranial tumors (eg. craniopharyngiomas), and hypersensitivity reactions [1,3]. In the majority of cases of persistent neutrophilic meningitis due to an infectious etiology, the syndrome is part of a multi-system disseminated process usually in an immunocompromised individual. The atypical nature of many of the organisms associated with this syndrome leads to difficulty in obtaining microbiologic diagnoses, and the etiologic agent is often presumed from epidemiologic risk factors, indirect microbiologic evidence or made postmortem [1,3]. Thus, the choice of empiric antibiotic treatment regimen can be a challenging clinical decision. Here we present a case of persistent neutrophilic meningitis in an immunocompetent individual following basilar skull fracture.

## Case Presentation

### Hospitalization 1 (basal skull fracture)

A 47 year-old healthy female sustained a ground level fall from a stepladder in her garage without loss of consciousness. The fall resulted in a basilar skull fracture complicated by a small subdural hematoma. She quickly recovered with conservative management and was discharged from the hospital within 4 days.

### Hospitalization 2a (acute meningitis at outside facility)

One week after her fall, she represented to an outside hospital with a fever of 102°F, headache, and altered mental status. Broad-spectrum antibiotics were initiated and after several days a lumbar puncture (LP) was performed. Analysis of the CSF was notable for an opening pressure of 33 cm of H_2_O, 3600 white blood cells (WBC) per deciliter with a neutrophilic predominance (Hospitalization 2a Table 1). CSF gram stain and cultures were obtained which demonstrated no organisms or growth, respectively. The patient eventually required a lumbar drain to manage persistently high intracranial pressures. A CT scan of the head and an MRI of the brain and spine revealed a small subdural hematoma without evidence of a para-spinal or para-meningeal focus of infection. Repeat analyses of the CSF demonstrated negative gram and silver stains, negative direct antigen testing for crytpococcus, *Hemophilus influenzae*, Influenza A and B, *Neisseria meningitidis, Streptococcus agalactiae *and *Streptococcus pneumoniae*; as well as bacterial and fungal cultures with no growth. Serologic testing for coccidiodimycosis, HIV and anti-nuclear antibody testing were negative. While on vancomycin and ceftazidime (14 days), she continued to have daily fevers associated with severe headaches, nausea, photophobia and neck stiffness. Due to a persistence of symptoms, ceftazidime was changed to meropenem and therapy continued for 14 additional days. A short period of defervescence occurred after the initiation of meropenem, but then the fever quickly returned while still on therapy. The patient was transferred to our facility for a second opinion.

**Table 1:** CSF analysis from the four hospitalizations noting neutrophilic predominance

	WBCs (cells/mm^3^)	Neutrophils (% of WBCs)	Lymphocytes (% of WBCs)	Glucose (mg/dL)	Total Protein (mg/dL)
Hospitalization 2a					
12/21	3600	83		32	33

Hospitalization 2b					
1/8	1850	83	11	21	90
1/13	2100	77	15	24	99

Hospitalization 3					
2/6	520	64	24	26	101
2/14	396	94	4	13	161

Hospitalization 4	480	83	14	17	

CSF = cerebral spinal fluid, mg/dl = milligram/deciliter, WBCs = white blood cells

### Hospitalization 2b (persistent neutrophilic meningitis, transfer to our facility)

On evaluation at our institution the patient was ill appearing and nauseated with a severe frontal headache that radiated to the occiput. Physical exam was notable for a temperature of 102°F and meningismus with an otherwise normal neurologic exam. A sutured laceration on the occipital region of the patient's head was noted to be moderately tender, but without fluctuance or drainage. Serially sampled CSF continued to have a predominantly neutrophilic pleocytosis (Hospitalization 2b Table 1). General labs were normal and repeated microbiologic studies were nondiagnostic. Broad-spectrum antibiotics were resumed (vancomycin and meropenem), however the patient continued to have high daily fevers. High dose trimethoprim-sulfamethoxazole (TMP-SMX) was added on the 8^th ^hospital day. An MRI of the brain and spine was notable for opacification of the left sphenoid sinus and mild pachymeninigitis at the occipital and anterior aspects of the brain; sites where coup and contrecoup injuries could be expected following the patient's fall. No focus of infection was identified. A non-displaced left lateral wall sphenoid sinus fracture and occipital bone fracture were noted on CT sinus scan. A follow up CT cisternogram was negative for any CSF leak. Upon endoscopic ethmoid and sphenoid sinus visualization, a sphenoid sinus hematoma was identified and removed. Bacterial, mycobacterial and fungal cultures of this sampled material remained negative for any growth. The patient's clinical course improved, and after six weeks of vancomycin and meropenem (including five days of TMP-SMX) antibiotics were serially discontinued and the patient was discharged home without an etiologic diagnosis.

### Hospitalization 3 (re-admission neutrophilic meningitis)

Nine days after discharge, the patient represented to our institution with fevers, headaches and lateral gaze ophthalmoplegia. Repeat LP and imaging studies were performed. The previously noted pachymeningitis and opacification of left sphenoid sinus and left occipital bone fracture were noted with no new findings. A tagged white blood cell (WBC) scan utilizing Indium-111 labeled WBC was also unrevealing. The patient underwent repeat endoscopic surgery with application of fibrin glue to the left sphenoid sinus (where the hematoma had been previously removed). During this procedure a pressurized fluorescein CSF dye study was performed which did not show any evidence of a CSF leak. The patient was treated with 14 days of meropenem and TMP-SMX therapy, and discharged home with clinical improvement, but no microbiologic diagnosis.

### Hospitalization 4 (re-admission neutrophilic meningitis)

Eleven days after completing meropenem and TMP-SMX, she once again developed fevers and headaches requiring a 4^th ^hospitalization at another institution. Eight weeks after sampling, the CSF culture from the 3rd hospitalization grew *Nocardia *spp. that eventually was identified as *Nocardia cyriacigeorgica *complex. The same organism was concomitantly recovered from a lumbar puncture performed during the 4^th ^hospitalization. The isolate was found to be sensitive to ceftriaxone, imipenem, linezolid, doxycycline, moxifloxacin and trimethoprim-sulfamethoxazole (Table 2). Ceftriaxone 2g IV daily and trimethoprim-sulfamethoxazole 2 DS BID were initiated after which the fevers, headaches and ophthalmoplegia resolved. The induction course of therapy was continued for 6 weeks followed by maintenance therapy with TMP-SMX 2 DS BID and doxycycline 100mg po bid for 1 year. As of the writing of this manuscript she is greater than 12 months post completion of therapy and remains asymptomatic.

**Table 2:** Sensitivity of *Nocardia cryiacigeorgica *isolated from the CSF

Organism:	Antibiotic	Sensitivity (MIC in mcg/ml)
*Nocardia cryiacigeorgica*	Ceftriaxone	1.2 (S)
-Isolated from CSF 2/14	Doxycycline	.6 (S)
	Imipenem	.3 (S)
	Linezolid	.6 (S)
	Moxifloxacin	2.5 (S)
	Trim/Sulfa	.3 (S)

CSF = cerebral spinal fluid

*Nocardia *is a gram-positive actinomycete that is a ubiquitous saprophyte found in the environment (soil, dust, decaying vegetation) [4]. It causes a wide spectrum of disease in humans depending on the immune status of the host. Primarily, it is an opportunistic pathogen that affects patients with impaired cell mediated immunity [5]. Infection with *Nocardia *most commonly presents as a pulmonary syndrome with nodules, necrosis and/or cavities in 77% of cases. Central nervous system infection in the form of brain abscesses [6] and occasionally meningitis [7,8] typically occurs after dissemination. In immunocompetent individuals, direct skin inoculation can result in skin and soft-tissue infections. Presentations of this form of nocardiosis include mycetoma, sporotrichoid infections and occasionally osteomyelitis [9,10].

Persistent neutrophilic meningitis is different from the usual forms of chronic meningitis that typically begin as a neutrophilic process but then evolve to a lymphocytic predominance. Several unique features of *Nocardia *enable neutrophilic persistence. High levels of catalase and superoxide dismutase produced by *Nocardia *species neutralize the killing capacity of neutrophils. T-cells and macrophages thus play an important role in clearing *Nocardia *infection, while neutrophils mainly act to inhibit filament formation and growth of the bacterium [11-13], The short half-life of the neutrophil requires continued neutrophil recruitment to maintain inhibitory effects on the pathogen's growth, which drives abscess formation [13]. Mouse models have demonstrated that the outcome of *Nocardia *infection (clearance vs. abscess formation) appears to be dependent on inoculum size, stage of *Nocardia *growth, and immune status of the mouse [14]. While *Nocardia *is a well-described cause of persistent neutrophilic meningitis [1,3], it generally occurs in immunocompromised patients following dissemination from a pulmonary focus. Here we present a case of chronic *Nocardia *meningitis in an immunocompetent host. We speculate that in our patient, a low inoculum of *Nocardia *entered the immune-privileged subdural space from the basilar skull fracture, resulting in prolonged infection with persistent neutrophilic inflammation without abscess formation.

This case highlights a number of the unique features of *Nocardia *meningitis including the acute to chronic presentation with fevers, headache and meningismus with persistent neutrophilic pleocytosis, hypoglycorrhachia and elevated CSF protein levels. The largest review of *Nocardia *meningitis includes 21 cases in which a predisposing condition was noted in 75% of cases, neutrophilic pleocytosis in 85%, hypoglycorrhachia in 64% and elevated protein in 61%. Forty-three percent of the cases also had a brain abscess and one case had a basilar skull fracture. Diagnosis was often difficult and often delayed likely contributing to high mortality. In this series, mortality was 57% overall [8] which is similar to other smaller case series [7]. The widespread clinical application of ribosomal DNA sequencing technology (16s rDNA) may lead to earlier diagnosis resulting in improved outcomes in these difficult cases. We cannot be certain in our patient if the portal of entry was direct inoculation from the occipital wound or from a sinus defect created by the basilar skull fracture with prior nasopharyngeal colonization.

Prior to molecular phenotyping *Nocardia asteroides *complex was taxonomically categorized into 6 drug pattern susceptibilities [15]. It is currently thought that *Nocardia cyriacigeorgica *(the pathogen recovered in this case) represents *Nocardia asteroides *drug pattern VI [16], and is the most common *Nocardia *spp isolate found in the southern United States [16]. The organism is typically susceptible to cephalosporins, amikacin, imipenem and linezolid, while resistant to penicillin, clarithromycin and ciprofloxacin [17]. Isolates of *Nocardia cyriacigeorgica *have recently been reported in a variety of clinical scenarios [18-20] making this organism an important clinical pathogen, which is increasingly being reported as a source of infection in the United States [17].

## Conclusions

This unique case demonstrates the importance of considering *Nocardia *infection as a cause of persistent neutrophilic meningitis even in immunocompetent patients. Microbiologic diagnosis can be difficult to obtain due to the organism's slow rate of growth and sensitivity to numerous antibiotics which are generally started early and empirically. Interestingly, our patient did clinically respond to treatment with meropenem, which was enough to sterilize the CSF but insufficient to fully treat the infection (as demonstrated by relapse even after a 6+ week course of therapy). Our patient eventually made a full recovery after completing 6 weeks of ceftriaxone and TMP-SMX followed by one year of therapy with TMP-SMX and doxycycline.

## Competing interests

The authors declare that they have no competing interests.

## Authors' contributions

JSG was directly involved in the care and diagnosis of this patient, manuscript preparation, editing and submission. SRA was involved in patient care, diagnosis and manuscript editing. DZU contributed to patient care, diagnosis and manuscript editing. SRM contributed to diagnosis, long term follow-up and patient care, manuscript preparation and editing. All authors have read and approved the final manuscript.

## Pre-publication history

The pre-publication history for this paper can be accessed here:

http://www.biomedcentral.com/1471-2334/11/136/prepub
